# Bioelectrical impedance vector analysis (BIVA) in sport and exercise: Systematic review and future perspectives

**DOI:** 10.1371/journal.pone.0197957

**Published:** 2018-06-07

**Authors:** Jorge Castizo-Olier, Alfredo Irurtia, Monèm Jemni, Marta Carrasco-Marginet, Raúl Fernández-García, Ferran A. Rodríguez

**Affiliations:** 1 INEFC-Barcelona Sport Sciences Research Group, National Institute of Physical Education of Catalonia (INEFC), University of Barcelona (UB), Barcelona, Spain; 2 Catalan School of Kinanthropometry, National Institute of Physical Education of Catalonia (INEFC), University of Barcelona (UB), Barcelona, Spain; 3 Department of Sport Science, Qatar University, Doha, Qatar; 4 Department of Electronic Engineering, Polytechnic University of Catalonia, Barcelona, Spain; Universite de Nantes, FRANCE

## Abstract

**Background:**

Bioelectrical impedance vector analysis (BIVA) is a general concept that includes all methodologies used in the analysis of the bioelectrical vector, whereas the "classic" BIVA is a patented methodology included among these methods of analysis. Once this was clarified, the systematic review of the literature provides a deeper insight into the scope and range of application of BIVA in sport and exercise.

**Objective:**

The main goal of this work was to systematically review the sources on the applications of BIVA in sport and exercise and to examine its usefulness and suitability as a technique for the evaluation of body composition, hydration status, and other physiological and clinical relevant characteristics, ultimately to trace future perspectives in this growing area, including a proposal for a research agenda.

**Methods:**

Systematic literature searches in PubMed, SPORTDiscus and Scopus databases up to July, 2017 were conducted on any empirical investigations using phase-sensitive bioimpedance instruments to perform BIVA within exercise and sport contexts. The search included healthy sedentary individuals, physically active subjects and athletes.

**Result:**

Nineteen eligible papers were included and classified as sixteen original articles and three scientific conference communications. Three studies analysed short-term variations in the hydration status evoked by exercise/training through whole-body measurements, eleven assessed whole-body body composition changes induced by long-term exercise, four compared athletic groups or populations using the whole-body assessment, and two analysed bioelectrical patterns of athletic injuries or muscle damage through localised bioimpedance measurements.

**Conclusions:**

BIVA is a relatively new technique that has potential in sport and exercise, especially for the assessment of soft-tissue injury. On the other hand, the current tolerance ellipses of “classic” BIVA are not a valid method to identify dehydration in individual athletes and a new approach is needed. “Specific” BIVA, a method which proposes a correction of bioelectrical values for body geometry, emerges as the key to overcome “classic” BIVA limitations regarding the body composition assessment. Further research establishing standardised testing procedures and investigating the relationship between physiology and the bioelectrical signal in sport and exercise is needed.

## Introduction

Bioelectrical impedance analysis (BIA) is a non-invasive technique widely used in body composition assessment [1–5], nutritional status [5–7], and hydration status [2, 8, 9], all considered areas of interest to monitor general health and well-being [10], but also training and performance levels. However, conventional BIA is limited by the use of models and algorithms that assume relations between body components are constant and correlated with each other during stable periods, which are used to estimate through simple or multiple regression equations an unknown body component from a related measured variable (bioimpedance) [11]. Multiple validation studies demonstrated solid relationship between bodily impedance and fluid volume (e.g. compared to isotope dilution), but their validity and accuracy of prediction are population-specific [12]. Furthermore, the standard errors of the best BIA regression equations were estimated to be, for instance, ~3–8% for total body water (TBW) and ~3–6% for fat-free mass (FFM), both considered too large to be used in clinical setting [12, 13]. In the exercise and sport practice, this is especially relevant. For example, dehydration processes lower than these standard errors which may affect negatively the sport performance could be not adequately detected [14].

BIA measures body tissues opposition to the flow of a low-level, alternating radiofrequency electric current. Bioelectrical impedance (Z)—i.e. the tissues opposition to the electric current flow—, the vector sum of the resistance (R)—i.e. the major resistance to the current through intra- and extracellular ionic fluids—and the reactance (Xc)—i.e. the additional opposition due to the capacitive elements such as cell membranes, tissue interfaces, and non-ionic substances. BIA has been performed using single- (SF-BIA) or multiple-frequency (MF-BIA) electrical current. Standard SF-BIA uses a single frequency of 50 kHz to estimate TBW and FFM, but does not differentiate intracellular water (ICW) and extracellular water (ECW), respectively. In an attempt to overcome this, MF-BIA tries to estimate ICW and ECW by measuring a spectrum of frequencies through different mathematical models [12]. However, MF-BIA models have significant limitations, such as the required use of body mass (BM) as an independent variable. Most scientific evidence shows that the use of both SF-BIA and MF-BIA lead to prediction errors in healthy people [5, 15–17] and even larger errors in people with clinical conditions [18, 19]. In spite of the widespread use of BIA in the clinical and field settings, mainly in the estimation of body composition, such as fat mass (FM) and FFM, or TBW, ICW and ECW, its accuracy is compromised because of its reliance on regression equations, mostly derived from non-athletic or sport-specific populations [5], and assumptions such as constant tissue isotropy or constant tissue hydration, conditions that are not frequently met [5, 11].

Alternative techniques such as the measure of the phase angle (PA) or the “classic” bioelectrical impedance vector analysis (“classic” BIVA) [20] emerged to overcome the above-mentioned BIA limitations, basing their main strength on the use of raw impedance parameters. It is important to mention that the present review distinguished between the term “classic” BIVA (commonly termed BIVA in the literature), the methodology patented by Pillon and Piccoli [21], and a more general concept that include all methodologies using vector analysis, i.e. bioelectrical impedance vector analysis (BIVA in the present review). This general concept include the whole-body assessment methods “classic” BIVA and “specific” BIVA (which is a methodology that tries to overcome some limitations of “classic” BIVA), and the localised bioelectrical impedance vector analysis (which is a method proposed for the identification and follow-up of muscle injuries). Once this was noted, it has to been clarified that “classic” BIVA does not provide quantitative estimates of tissue mass (kg) or fluid volumes (L). Instead, it is qualitative and semi-quantitative evaluation of body cell mass (BCM) and hydration [22, 23]. The number of publications using “classic” BIVA in clinical practice increased exponentially during the last decade due to its strengths [11, 18, 24–30]. Nowadays, “classic” BIVA is a widely used technique in medicine as a tool for the assessment of hydration and nutritional status (e.g. fluid imbalance and wasting of lean tissues, respectively) in different clinical conditions, such as renal disease [31], critically ill patients [32], obesity [33] and morbid obesity [34], pulmonary disease [30], anorexia nervosa [26], cachexia [25], sarcopenia and sarcopenic obesity [27], Alzheimer’s disease [29], heart failure [25], gastrointestinal disease [28], diabetes [24], wound healing [35], muscle injury assessment [36, 37], and pregnancy and postpartum [38]. Validation studies of “classic” BIVA have shown a significant association of bioelectrical values with hydration [11, 39], and nutritional status [11] in clinical conditions. Several studies have compared “classic” BIVA parameters with conventional BIA and other measures of body composition such as dual-energy X-ray absorptiometry (DXA), anthropometry, and clinical evaluation in samples of healthy and sick populations with mixed results (for review see [4, 11, 40]).

There has been a rapid growth of interest in the application of BIVA in sport and exercise research and practice in the recent years. On the one hand, “classic” BIVA is being used to characterise the body composition (i.e. hydration status and BCM) of athletes and active individuals [36, 41–43] and to monitor body composition longitudinal changes induced by exercise or sport practice [44–57]. On the other hand, the localised bioimpedance vector analysis is being applied for the identification and follow-up of muscle injuries [36, 37]. The importance of assessing the body composition of athletes lies in the fact that the physical stress imposed during trainings and competitions may lead to body composition alterations, which can be detrimental to athletes [58]. Furthermore, body composition has been suggested to discriminate athletes of different performance levels [59, 60] and has been shown to influence physical performance [61] and sport success [62]. On the other hand, the importance of monitoring the hydration status in exercise and sport is because dehydration is recognised to impair sport performance [63, 64], as well as increasing the injury risk [65]. Monitoring body fluid variations may help to adequately prescribe fluid intake and thus limit deleterious effects. Furthermore, the identification of injury and its follow-up during recovery until return-to-play depends on expensive methods, which are not accessible to everyone. Therefore, the increase in the number of publications regarding BIVA in the exercise and sport field seems justified in order to investigate the applicability of the method for assessments in real time and in a precise, accurate, reliable, non-invasive, portable, inexpensive, safe and simple way. In addition, since the current scientific literature in this field is still scarce and very heterogeneous, a compilation of the current knowledge is needed in order to suggest a research agenda.

### Objectives

This systematic review aims to summarise the current knowledge on the applications of BIVA in sport and exercise, and to evaluate the usefulness and suitability of the method in assessing body composition, hydration status, and other physiological and clinical conditions in healthy sedentary people, physically active and trained individuals. Ultimately, this review attempts to outline future perspectives in this field and to suggest a research agenda.

## Methods

Preferred Reporting Items for Systematic Reviews and Meta-Analyses (PRISMA) guidelines were applied to undertake the present review [66]. PRISMA checklist was also used to elaborate the systematic review protocol [67].

### Eligibility criteria

This study reviewed and analysed methodological, clinical, and empirical studies using phase-sensitive devices to perform the analysis within the context of exercise and sport. The phase-sensitivity characteristic is important since non phase-sensitive instruments do not measure Xc, and the proper way to apply BIVA needs both R and Xc. Articles that have used BIVA in healthy sedentary people, physically active individuals and athletes of all levels were eligible for review. Studies were screened for eligibility on the following inclusion criteria: (a) empirical investigations with BIVA measures taken in human subjects performing acute or chronic exercise; (b) empirical investigations with BIVA measures taken in healthy sedentary people, physically active individuals and athletes; c) studies where data acquisition was performed with the appropriate methodology; (d) studies published in a peer-reviewed journal and/or in relevant congress proceedings; and (e) studies published in English language. No restrictions in terms of study design, setting, country or time frame were considered.

### Information sources

**Fig 1:**
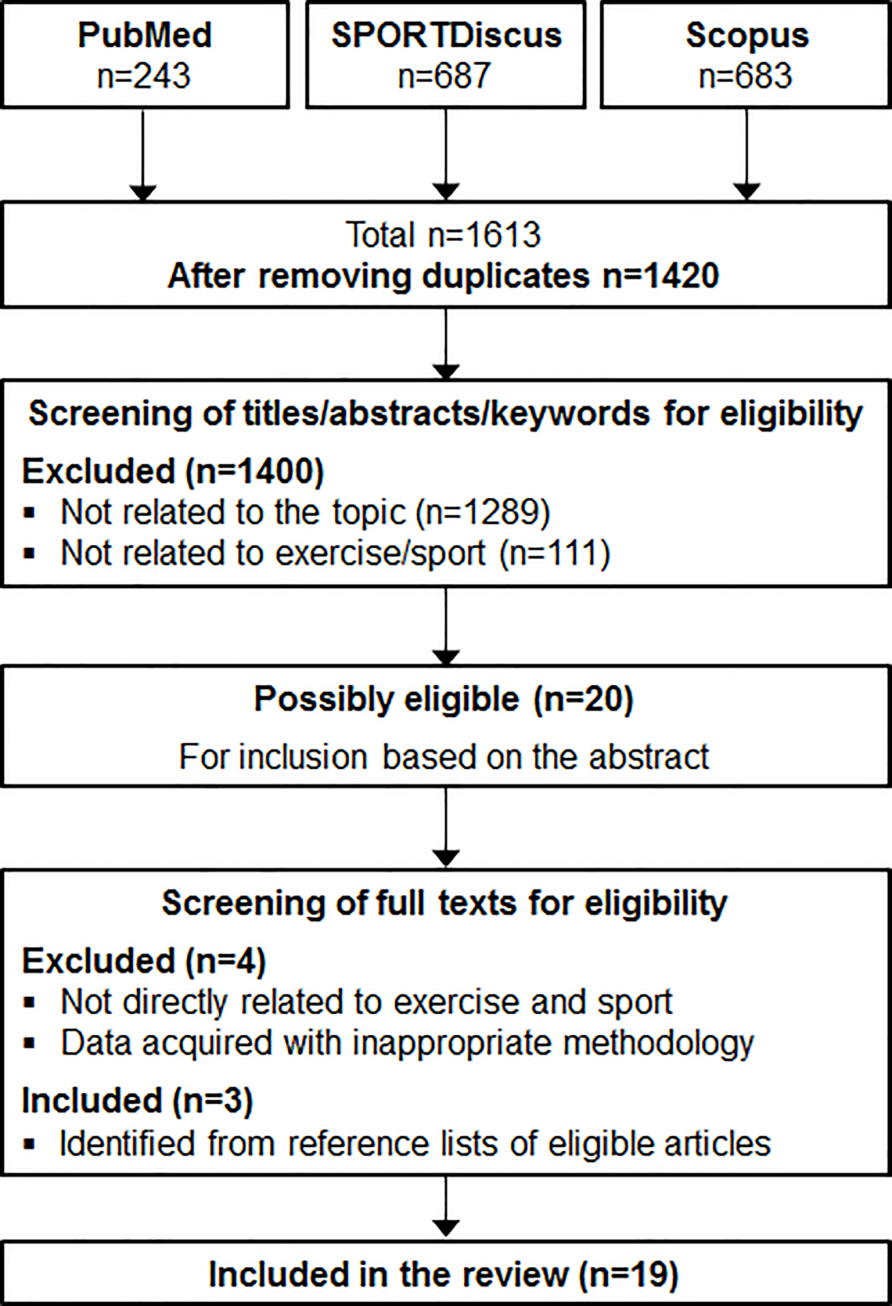
Flow chart of study identification and eligibility for the systematic review.

A computer-based literature search was conducted for the period 1994–2017, ending by July 2017, of PubMed, SPORTDiscus and Scopus databases (Fig 1).

### Search strategy

Title, abstract, and keyword fields were searched in each of the aforementioned databases using the following search terms and syntax: (“BIVA” OR “vector*”) AND (“hydration” OR “body water”).

### Study records

Records were exported from the electronic databases to a reference management software (EndNote, v. X5, Thomson Reuters, 2011) and duplicate references were removed. Fig 1 displays the flow chart of study identification and eligibility for the systematic review.

The eligible articles after removing duplicates were screened by two investigators (JCO, AI), with disagreement settled by consensus. An initial screening of titles, abstracts and keywords was performed in order to check for inclusion criteria and to exclude obviously irrelevant records using the eligibility criteria (Fig 1). Differences in study eligibility for review were compared and deviations were discussed with a third investigator (FAR) until consensus could be reached. When a paper could not be rejected with certainty it was included in the eligible papers for full text evaluation. Then, articles were assessed for eligibility through a full-text screening, and those meeting the established criteria were included in the review. The reference lists of articles retrieved for inclusion in the review up to this point were searched to identify other relevant investigations. The number of studies meeting the pre-specified inclusion criteria and those excluded and reasons for their exclusion were recorded (Fig 1).

Each selected article was reviewed for information on (1) bibliographic characteristics (type of publication, authors, year and journal); (2) aims of the investigation; (3) study design and methodology; (4) sample characteristics (number, population, gender, age, exercise activity, sport discipline, and sport competitive level of subjects); (5) BIA device employed; (6) electrode distribution; (7) BIVA approach (whole-body BIVA or localised bioimpedance vector analysis); (8) vector displacement and (9) comparative technique (e.g. other indicators to assess body composition and fluid status, injury assessment).

### Data items and prioritisation

Full texts were reviewed in search for the following main variables: bioelectrical resistance (R, R/h), reactance (Xc, Xc/h), Z, PA, RXc graph, TBW, ICW, ECW, FM, FFM and BCM. Bioelectrical measures and directly derived parameters were considered the main outcome from the population studies or experimental interventions. From a methodological point of view, comparisons of BIVA outcomes with other measures of body composition and fluid status assessment could underpin the validity of the technique and, therefore, the latter were considered additional outcomes.

## Results

### Search outcome

After removal of duplicates, 1420 records were identified, which were reduced to 20 after screening titles, abstracts and keywords for eligibility (Fig 1). After full-text evaluation, 19 studies matched the selection criteria and were included in the qualitative synthesis analysis and summarised in Tables 1–4. Table 5 compiles the information about the baseline bioelectrical parameters and vector position of the participants analysed in the studies included in the present review.

**Table 1:** BIVA studies analysing short-term changes (<24 hours) in the hydration status induced by exercise and training.

Study	Publication	Aim	Design	Methodology	n	Sex	Age	Sport/Exercise	Level	BIA device	Electrode distribution	Vector/BIA differences (Yes / No)*	Comparative technique
Gatterer et al. 2014 [47]	Original article	To analyse bioelectrical changes induced by exercise under heat stress (environmental chamber) with hydration biomarkers	Short-term vector changes (1 h of exercise)	Analysis of intra-individual and intra-group differences. Comparison with the healthy reference population	14	M	24.1±1.7	Self-rated intensity (Borg Scale) cycle ergometer test	Well trained subjects	BIA 101 ASE, Akern/RJL (P-S)	Whole-body	Yes	Directional changes in vector values towards the upper pole of the ellipses occurred along with BM and plasma osmolality changes after exercise
Antoni et al. 2017 [51]	Original article	To analyse bioelectrical changes induced by a subterranean exploration	Short-term vector changes (~10 h of physical activity)	Analysis of intra-group and inter-group differences. Comparison with the healthy reference population	40	F, M	44.0±19	Caving	Beginners, amateurs and experts	BIA 101 ASE, Akern/RJL (P-S)	Whole-body	Yes (Xc and PA, only in men)	Directional changes in vector values towards the upper pole of the ellipses occurred along with a significant increase in BM in the group of men
Carrasco-Marginet et al. 2017 [57]	Original article	To analyse bioelectrical changes induced by a synchronised swimming training	Short-term vector changes (~2.5–3.5 h of exercise)	Analysis of intra-group and inter-group differences. Comparison with the healthy reference population	49	F	Pre-junior (n = 34): 13.9±0.9 Junior (n = 15): 16.3±0.6	Synchronised swimming	Elite	Z-Metrix, Bioparhom (P-S)	Whole-body	Yes	Directional changes in vector values towards the upper pole of the ellipses and significant mean vector differences occurred along with BM changes after exercise

M: males; F: females; P-S: phase-sensitive device; BIA: bioelectrical impedance analysis; BIVA: bioelectrical impedance vector analysis; Xc: reactance; h: height; BM: body mass

* Significance level: p<0.05

**Table 2:** BIVA studies analysing long-term (≥7 days) changes in body composition induced by exercise and training.

Study	Publication	Aim	Design	Methodology	N	Sex	Age	Sport/Exercise	Level	BIA device	Electrode distribution	Vector/BIA differences (Yes / No)*	Comparative technique
Piccoli et al. 1996 [49]	Original article	To analyse bioelectrical changes induced by a high altitude climbing expedition	Long-term vector changes (~12 weeks)	Analysis of intra-individual and intra-group differences. Comparison with the healthy reference population	7	M	25 (22–28)	Climbing	Healthy subjects	BIA-101, Akern/RJL Systems (P-S)	Whole-body	Yes	Bioelectrical changes correlated with changes in BM and hydration biomarkers
Gatterer et al. 2011 [46]	Original article	To analyse bioelectrical changes induced by two soccer matches	Long-term vector changes (~1–2 weeks)	Analysis of intra-group differences. Comparison with the healthy reference population	14	M	Starters (n = 7): 24.3±3.0 Non-starters (n = 7): 26.0±5.0	Soccer	Elite	BIA 2000-M, Data Input GmbH (P-S)	Whole-body	Yes	Significant vector displacement along with BM changes were observed in the starters group between the first and the second match
Bonuccelli et al. 2011 [44]	Scientific congress communication	To analyse bioelectrical changes induced by a soccer season	Long-term vector changes (whole season)	Analysis of intra-group differences	18	M	27.6±4.9	Soccer	Elite	BIA-101, Akern/RJL Systems (P-S)	Whole-body	Yes	No comparative technique was reported
Bonuccelli et al. 2012 [45]	Scientific congress communication	To analyse bioelectrical and DXA changes induced by a soccer season	Long-term vector changes (whole season)	Analysis of intra-group differences	10	M	26.7±3.0	Soccer	Elite	BIA-101, Akern/RJL Systems (P-S)	Whole-body	Yes	BIVA was sensitive to body composition changes (identified by DXA) through a soccer season
Reljic et al. 2013 [50]	Original article	To analyse bioelectrical changes with hydration biomarkers	Long-term vector changes (unspecified duration)	Analysis of intra-group differences. Comparison with the healthy reference population	17	M	Weight-loss group (n = 10): 19.7±3.2 Control (n = 7): 18.4±2.2	Boxing	Elite	BIA-101, Akern/RJL Systems (P-S)	Whole-body	Yes	Directional changes in vector values towards the upper pole of the ellipses occurred along with significant changes in BM and blood parameters within few days before competition
Mascherini et al. 2014 [48]	Original article	To analyse bioelectrical changes induced by a soccer season	Long-term vector changes (whole season)	Analysis of intra-group differences. Comparison with the soccer specific reference population	18	M	21.8±3.0	Soccer	Professional	BIA-101 ASE, Akern/RJL Systems (P-S)	Whole-body	Yes	Changes in the vector length correlated with changes in the endurance performance
Mascherini et al. 2015 [54]	Original article	To analyse bioelectrical changes induced by a soccer training program	Long-term bioelectrical changes (50 days)	Analysis of intra-group differences. Comparison with the healthy reference population	59	M	22.5±5.6	Soccer	Elite	BIA-101 ASE, Akern/RJL Systems (P-S)	Whole-body and localised	Yes	Bioelectrical differences in the whole-body and localised assessments were found along with some anthropometric measures changes after 50 days of training
Fukuda et al. 2016 [52]	Original article	To analyse bioelectrical changes induced by a resistance training program	Long-term vector changes (6 months)	Analysis of intra-group differences	20	F	71.9±6.9	Full-body resistance training program	Healthy, ambulatory subjects	Quantum II, RJL Systems (P-S)	Whole-body	Yes	Significant training effects were found for PA after the training program. No relationship was observed between changes in strength and BIA after 6 months.
Pollastri et al. 2016 [55]	Original article	To analyse bioelectrical changes induced by a multistage road bicycle race (Giro d'Italia 2014)	Long-term vector changes (3 weeks)	Analysis of intra-group differences. Comparison with the healthy reference population	9	M	28.2±4.7	Cycling	Professional	BIA-101 ASE, Akern/RJL Systems (P-S)	Whole-body	Yes	BIA vector changes were not related to power output or RPE
Pollastri et al. 2016 [56]	Original article	To analyse bioelectrical changes induced by a multistage road bicycle race (Giro d'Italia 2014)	Long-term vector changes (3 weeks)	Analysis of intra-group differences	8	M	28.8±4.7	Cycling	Elite	BIA-101 ASE, Akern/RJL Systems (P-S)	Whole-body	Yes	BIA vector changes correlated with maximal mean power of different time durations depending on the stage
Meleleo et al. 2017 [53]	Original article	To analyse bioelectrical changes induced by daily competitive sport	Long-term vector changes (1 year)	Analysis of intra-group and inter-group differences	219	F, M	Non-athletic group: 9.3 (8.2–10.5) Athletic group: 9.5 (8.0–10.5)	Swimming Gymnastics	Healthy subjects	BIA-101 ASE, Akern/RJL Systems (P-S)	Whole-body	Yes	Bioelectrical differences were found along with a lack of difference in BMI between groups

M: males; F: females; P-S: phase-sensitive device; BIA: bioelectrical impedance analysis; BIVA: bioelectrical impedance vector analysis; DXA: dual-energy X-ray absorptiometry; R: resistance; Xc: reactance; PA: phase angle; h: height; BM: body mass; RPE: rating of perceived exertion; BMI: body mass index

* Significance level: p<0.05

**Table 3:** BIVA studies analysing bioelectrical differences between populations.

Study	Publication	Aim	Design	Methodology	N	Sex	Age	Sport/Exercise	Level	BIA device	Electrode distribution	Vector/BIA differences (Yes / No)*	Comparative technique
Piccoli et al. 2007 [42]	Original article	To assess the equivalence of information between BIA (50 kHz) and BIS in two different groups	Single measure	Inter-group analysis. Comparison with the healthy reference population	60	M	Bodybuilders (n = 30): 32.1±5.7 Controls (n = 30): 25.2±5.3	Bodybuilding	Professional	SEAC SFB3, UniQuest-SEAC (P-S); BIA-101, RJL Systems (P-S)	Whole-body	Yes	R and Xc (50 kHz) correlated with other frequencies. Estimated TBW with BIS correlated with Sun's formula (50 kHz)
Micheli et al. 2014 [41]	Original article	To assess BIVA in soccer players and establish new specific tolerance ellipses	Single measure	Inter-group analysis. Comparison with the healthy reference population	893	M	24.1±5.1	Soccer	Elite and professional	BIA-101, Akern/RJL Systems (P-S)	Whole-body	Yes	Elite and high-level soccer players registered significant bioelectrical and BM differences compared with lower performance levels
Koury et al. 2014 [43]	Original article	To assess BIVA in adolescent and adult athletes	Single measure	Inter-group analysis. Comparison with the healthy reference population	195	M	Adolescents (n = 105): 15.1±2.1 Adults (n = 90): 28.9±7.3	Athletics (n = 25) Soccer (n = 50) Swimming (n = 22) Water polo (n = 15) Triathlon (n = 20) Basketball (n = 20) Adventure running (n = 6) Cycling (n = 15) Marathon (n = 15) Judo (n = 7)	Elite	Quantum BIA-101Q, RJL-101 (P-S)	Whole-body	Yes	PA correlated with BM and age

M: males; F: females; P-S: phase-sensitive device; BIA: bioelectrical impedance analysis; BIVA: bioelectrical impedance vector analysis; BIS: bioelectrical impedance spectroscopy; R: resistance; Xc: reactance; PA: phase angle; BM: body mass; TBW: total body water

* Significance level: p<0.05

**Table 4:** BIVA studies analysing bioelectrical changes induced by injury.

Study	Publication	Aim	Design	Methodology	N	Sex	Age	Sport/Exercise	Level	BIA device	Electrode distribution	Vector/BIA differences (Yes / No) *	Comparative technique
Nescolarde et al. 2011 [36]	Scientific congress communication	To analyse whole-body and localised bioelectrical differences between two sports, and to assess muscle injuries	Single measure	Inter-group analysis. Comparison with the healthy reference population	14	M	>18.0	Soccer (n = 10) Basketball (n = 4)	Professional	BIA-101, Akern-RJL Systems (P-S)	Whole-body and localised	Yes	Localised BIA was sensitive to different types of injury diagnosed by magnetic resonance imaging
Nescolarde et al. 2013 [37]	Original article	To analyse bioelectrical changes induced by injury and its recovery	Long-term bioelectrical changes (9 to 75 days)	Analysis of intra-individual differences (injury identification and follow-up)	3	M	22.0±3.6	Soccer	Professional	BIA-101, Akern/RJL Systems (P-S)	Localised	Yes	Localised BIA was consistent with reference magnetic resonance imaging diagnoses with differing levels of injury severity

M: males; P-S: phase-sensitive device; BIA: bioelectrical impedance analysis; BIVA: bioelectrical impedance vector analysis

* Significance level: p<0.05

**Table 5:** Baseline bioelectrical parameters and vector position of the participants analysed in the studies included in the present review.

Study	BMI (kg/m^2^)	R/h (Ω/m)	Xc/h (Ω/m)	PA (º)	Vector position on the BIVA point graph	Other comments
Nescolarde et al. 2011 [36]	Soccer: 23.2±1.5 Basketball: 24.3±1.1	Soccer: 268.9±22.4 Basketball: 221.8±22.9	Soccer: 37.4±3.8 Basketball: 28.8±4.9	Soccer: 7.9±0.7 Basketball: 7.4±0.6	Soccer: The mean vector was plotted inside the “athlete” quadrant of the reference population, outside the range of normal hydration Basketball: The mean vector was plotted inside the “obese” quadrant of the reference population, outside the range of normal hydration	
Nescolarde et al. 2013 [37]	NR	NR	NR	NR	NR	
Micheli et al. 2014 [41]	All: 23.3±1.6	All: 263.9±26.2	All: 33.8±3.9	All: 7.3±0.6	The individual vectors were scattered in both “athlete” and “obese” quadrants of the reference population, outside and inside the range of normal hydration	Some individual vectors were plotted inside the “lean” quadrant of the reference population, outside and inside the range of normal hydration
Piccoli et al. 2007 [42]	BB: 28.9±3.6	BB: NR	BB: NR	BB: 8.6±1.1	The mean vector was plotted in the limit of the 95% ellipse of the “obese” quadrant of the reference population, outside the range of normal hydration	
Koury et al. 2014 [43]	Adolescent: 20.2±3.0 Adult: 22.7±2.7	Adolescent: 302.0±71.0 Adult: 252.4±33.8	Adolescent: 36.2±6.7 Adult: 35.4±4.9	Adolescent: 6.9±0.9 Adult: 8.0±0.7	Adolescent: The majority of the individual vectors were scattered inside the “obese” quadrant of the reference population, either when all the participants were plotted and when the comparison was performed according to paired sport modalities. Most of them were plotted outside the range of normal hydration Adult: The majority of the individual vectors were scattered in both “athlete” and “obese” quadrants of the reference population, either when all the participants were plotted and when the comparison was performed according to paired sport modalities. Most of them were plotted outside the range of normal hydration	
Bonuccelli et al. 2011 [44]	NR	NR	NR	NR	NR	
Bonuccelli et al. 2012 [45]	NR	NR	NR	NR	NR	
Gatterer et al. 2011 [46]	S: 23.5±0.9 NS: 24.3±1.1 All: 23.9±1.1	NR	NR	NR	The mean vectors of both groups were plotted inside the “obese” quadrant of the reference population, close to the “athlete” one, outside the range of normal hydration	
Gatterer et al. 2014 [47]	NR	284.1±23.0	37.5±3.3	NR	Mean and individual vectors were plotted inside the “athlete” quadrant of the reference population, the majority of them outside the range of normal hydration	Only one individual vector was plotted inside the “obese” quadrant of the reference population, close to the “athlete” area, outside the range of normal hydration
Mascherini et al. 2014 [48]	NR	272.7±24.9	36.0±4.0	7.5±0.5	The mean vector was plotted inside the “lean” quadrant of the reference population, within the range of normal hydration	
Piccoli et al. 1996 [49]	22.9 (21.8–25.6)	256.5	31.2	NR	The mean vector was plotted inside the “obese” quadrant of the reference population, in the limit of the range of normal hydration	The article shows two examples of individual vectors, one plotted inside the “athlete” quadrant of the reference population (outside the range of normal hydration) and the other inside the “obese” one (within the range of normal hydration)
Reljic et al. 2013 [50]	NR	NR	NR	NR	The mean vectors of both groups were plotted inside the “athlete” quadrant of the reference population, within the range of normal hydration	
Antoni et al. 2017 [51]	F: 21.8±2.1 M: 24.7±3.0	F: 388.6±34.1 M: 296.6±38.5	F: 33.7± 3.2 M: 28.1± 5.9	F: 8.7± 0.8 M: 9.4± 1.3	F: The mean vector of women was plotted between the “cachexic” and the “lean” quadrants of the reference population, close to the left ones, within the range of normal hydration M: The mean vector of men was plotted inside the “cachexic” quadrant of the reference population, close to the “obese” one, within the range of normal hydration	
Carrasco-Marginet et al. 2017 [57]	Co: 18.0±1.9 Jr: 19.3±1.3 All: 18.4±1.8	Co: 328.4±38.8 Jr: 299.9±21.6 All: 319.7±36.7	Co: 40.0±4.5 Jr: 39.6±2.2 All: 39.9±3.9	Co: 7.0±0.5 Jr: 7.5±0.4 All: 7.1±0.5	Co: The majority of the individual vectors were plotted outside and inside the 95% tolerance ellipse of the “obese” quadrant of the reference population, outside the range of normal hydration Jr: The majority of the individual vectors were plotted outside the 95% tolerance ellipse of the “obese” quadrant of the reference population, outside the range of normal hydration. None of them were located inside the “athlete” quadrant	Some of the Co individual vectors were plotted inside the “athlete” quadrant of the reference population, most of them outside the range of normal hydration
Fukuda et al. 2016 [52]	24.5±3.0	376.9±45.4	31.6±5.5	4.8±0.6	NR	
Meleleo et al. 2017 [53]	F: 17.68 M: 19.68	F: 465.6±13.7 M: 418.7±14.9	F: 46.8±1.6 M: 40.6± 1.7	F: 5.8± 0.1 M: 5.6± 0.2	NR	
Mascherini et al. 2015 [54]	23.3±1.5	259.8±27.0	35.5±3.5	7.8±0.6	The mean vector was plotted inside the “athlete” quadrant of the reference population, outside the range of normal hydration	
Pollastri et al. 2016 [55]	NR	NR	NR	NR	NR	
Pollastri et al. 2016 [56]	NR	NR	NR	NR	Mean and individual vectors were plotted inside the “athlete” quadrant of the reference population, outside the range of normal hydration	

BMI: body mass index; R: resistance; Xc: reactance; h: height; PA: phase angle; BIVA: bioelectrical impedance vector analysis; NR: not reported; BB: bodybuilders; S: starters; NS: non-starters; Co: pre-junior; Jr: junior; F: females; M: males

The reviewed studies were sixteen original articles and three scientific conference communications. Publication date ranged from 1996 to 2017, yet only two studies were published before 2011, corroborating the novelty of the technique in the field of sport science.

### Participants

A total number of 1667 subjects participated in the different studies, yet most took part in a soccer population study (n = 893) [41] an athletic vs. non-athletic comparative investigation (n = 219) [53] and a multisport comparative research (n = 195) [43]. Most studies were performed in males and only four included females [51–53, 57]. Only three studies analysed non-adult populations [43, 53, 57]. Fourteen studies were carried out with elite or professional athletes.

### Finding outcomes

Three studies were aimed at analysing short-term changes (<24 hours) in the hydration status induced by exercise and training [47, 51, 57] (Table 1), eleven assessed body composition changes induced by exercise at the long term (≥ 7 days) [44–46, 48–50, 52–56] (Table 2), three compared athletic groups or populations [41–43] (Table 3), and two of the articles related bioelectrical patterns to athletic injury or muscle damage [36, 37] (Table 4).

### Bioelectrical measures

Most studies used whole-body electrode distribution, one used localised electrode distribution to analyse injury-induced bioelectrical changes [37], and two combined the standard whole-body and the localised techniques [36, 54]. The majority of the investigations used single-frequency impedance devices (50 kHz), two used multiple frequency bioimpedance analysers [46, 57] and one used both types of devices [42].

## Discussion

### BIVA applications in sport and exercise

#### Sporting population studies

These types of studies (Table 3) consist of single measure, cross-sectional protocols aiming to characterise sporting group samples in terms of bioelectrical data. As observed by Koury et al. [43], athletes exhibit similar trends of PA variation with age to those of the general population of the same sex and age, with a positive correlation (r = 0.63, p = 0.0004) in adolescents and a negative correlation (r = -0.27, p = 0.009) in adults. Vectors shifted to the left and with greater PA were found in both adolescent and adult athletes compared to the corresponding reference populations, which is consistent with the results reported by other studies for soccer players [41] and synchronised swimmers [57], suggesting that these differences are due to sport-specific adaptations [41]. In comparison with adolescent athletes, the mean vector of adult athletes also showed a shift to the left. Both shifts to the left indicate increased BCM and fluid content, and might reflect a better cell functioning [41].

Regarding the vector position on the RXc graph, the trend is to be outside the 50% tolerance ellipse of the respective reference population in both adolescent and adult athletes. According to this, Piccoli et al. [42] also found the mean impedance vector of bodybuilders almost completely outside the 95% tolerance ellipse of the reference population. This reflects a specific body composition and suggests that specific tolerance ellipses are needed for sport populations [36, 41, 57]. To date, only two studies [41, 57] have characterised sport-specific populations. The relationship between the new specific tolerance ellipses (for each sport, gender, age and race) and the hydration status, body composition and sport performance level should be analysed, in order to represent significant hydration changes (that compromise health or performance) or target zones of impedance vectors for athletes. Nevertheless, it is possible that a new approach is required for the exercise and sports field, beyond the current BIVA point graph, based on 50–95% tolerance ellipses and quadrants related to clinical outputs. With regard to the hydration assessment, it should be noted that fluid overload (overhydration) is not common in healthy athletes. Therefore, the analysis of the hydration status should be related to euhydration and physiological dehydration processes. In this way, as mentioned in Heavens et al. [68] regarding the identification of dehydration with single and serial measurements according to the tolerance ellipses of the reference population, the limits for “normal hydration” (individuals positioned within the 50% tolerance ellipses, according to the literature [18, 69]) should be reviewed, since subjects experiencing high levels of fluid loss can still be identified as euhydrated [68]. Other studies related to sport and exercise [47, 49] identified some individuals as euhydrated after significant BM decreases. Moreover, as shown in Table 5, the majority of the studies analysed identify the athletes outside the 50% tolerance ellipse. This is probably due to a range of “normal hydration” comprised by the ellipses wider than a hydration status/change considered as “dehydration” through other methodologies [68]. Nevertheless, the conclusions of Heavens et al. [68] should be confirmed with the appropriate methodology, since the study was not performed with a phase-sensitive device, and therefore, they could not obtain the real value of Xc. Therefore, although directional changes in vector values from serial measurements seem to be consistent with fluid loss, the current BIVA point graph is not a valid method to detect dehydration in individual athletes. Research investigating different levels of dehydration and their relationship with the new specific tolerance ellipses is needed in order to identify the limit of “normal hydration”. Furthermore, different types of dehydration can be experienced in sport: a) hypertonic dehydration (i.e. primarily a loss of water) is a common type of dehydration developed after exercise in which heavy sweating occurs; b) hypotonic dehydration (i.e. primarily a loss of electrolyte) and c) isotonic dehydration (i.e. equal losses of electrolytes and water), both may be developed by athletes competing in aesthetic-type sports and in weight classification sports in which fasting, vomiting and diuretic use are common behaviours [65]. Thus, research is needed related to the sensitivity of “classic” BIVA to each type of dehydration, as well as the behaviour of each one with regard to the tolerance ellipses. On the other hand, it should be investigated the relationship between the new specific tolerance ellipses and different sport performance levels. Maybe different sectors of the tolerance ellipses identify target zones for the athletes. With regard to the body composition assessment and in accordance with “classic BIVA”, athletes have been identified in the upper left quadrant of the reference population and obese individuals in the lower left quadrant. This would generally imply greater R/h and Xc/h values of the athletes. Nevertheless, as mentioned in the literature [22, 70], according to the electro-physical assumptions, FFM is characterised by a greater conductivity in comparison with the poorly hydrated adipose tissue, not justifying the relative shortness of vectors of obese individuals with respect to the athletes, unless contemplating their generally greater FM, fluid overload and body size. Furthermore, the vector position of athletes regarding the tolerance ellipses of the general reference population is controversial [4]. As mentioned by Buffa et al. [4], athletic individuals are not always plotted in the “athlete” quadrant of the reference population and their vectors often overlap the “obesity” area. This controversy can be observed in Table 5. From the nineteen investigations analysed, six studies did not report vectors distribution with regard to the reference population and only four found the majority or all the vectors of athletes positioned in the “athlete” area [47, 50, 54, 56]. Comparable vector position of athletes and obese individuals would imply similar values of R/h and Xc/h. The already mentioned factors FM and fluid overload could compensate the bioelectrical values between both individuals, not being “classic” BIVA (50 kHz) able to detect the differences (e.g. discriminating fluids distribution between compartments, with greater ICW content in athletes). Moreover, as mentioned in the literature [22, 70], “classic BIVA” would be characterised by a limited sensitivity in assessing the features of body composition (i.e. FM and FFM) due to the no consideration of the effect of cross-sectional areas of the body which interferes with bioelectrical values as well as lengths, according to the basic conductor theory (impedance is proportional to the conductor length and inversely related to its cross-sectional area) [71]. This effect of cross-sectional areas is particularly relevant in sport sciences because athletes of different disciplines generally differ in their body shape. To overcome this limitation of “classic” BIVA, a relatively new procedure (“specific” BIVA) has been developed [22, 70]. This method proposes a correction of bioelectrical values for body geometry and it has proven to be effective in identifying the relative proportion of FM in adults and elderly [22, 70]. Although the inclusion of anthropometric measurements can make these plots more sample-specific and perhaps less generalizable than “classic” BIVA, this adaptation may be an advance when comparing athletes with different body composition (in terms of FM and FFM). Therefore, it should be further investigated in the sports field.

Koury et al. [43] observed that the distance between the confidence ellipses of adolescent and adult athletes was lower than between the ellipses among their respective reference populations, either considering all sport modalities or only paired modalities. The authors speculated that the intense training reduced the differences between adolescent and adult individuals, although this is still to be elucidated. In their study, vector and PA differences were due to differences in R/h, significantly lower in adult athletes than in adolescent athletes, with no differences in Xc/h. Similar to these findings, Micheli et al. [41] reported that in soccer players of higher competitive level, vectors shifted to the left due to a decrease in R/h, with no difference in Xc/h compared to those in lower soccer divisions. This shift to the left was also found between elite and high-level players. As suggested by Micheli et al. [41], these results reflect different ICW content (adult > adolescent; higher > lower sport levels), since the ECW/TBW ratio is inversely related to PA [72], and it could be due to the hypertrophy of muscle fibres. Furthermore, despite similar training loads among players of the highest level, differences may be due to different individual responses to the training load, or they could also be an indicator of better training and/or recovery strategies in elite teams [41]. Carrasco-Marginet et al. [57] also reported a shift to the left, with no difference in Xc/h, in young synchronised swimmers of higher competitive level. Nevertheless, since higher-level swimmers were older than the lower-level ones, it should be investigated whether the differences were due to biological maturation, to specific training or a combination of both.

As noted, a greater PA accompanying a vector shifted to the left has been observed in adult athletes compared to the healthy reference population [36, 41–43, 46, 49, 50]. This is due to i) a decreased R/h as a result of a different body composition, probably due, among other factors, to a greater muscle mass, muscle glycogen reserves and plasma volume [73, 74], and ii) an increased Xc/h, probably due to an increase in the size and number of muscle cells (hypertrophy and hyperplasia, respectively), although the last one is still a controversial topic [75]. However, since a decreased R/h is also related to greater FM [33], further research is needed in order to clarify the reason for this behaviour. Furthermore, Xc/h is not only conditioned by the cell size, but also by the thickness and composition of the cell membranes and also by the distance between them, due to their relationship with membrane capacitance (Cm) [76]. In this way, lower Xc/h values have been documented in bodybuilders (the best model of extreme muscle hypertrophy) compared to healthy active people and with no differences with the healthy reference population [42]. However, vectors shifted to the left with lower PA have been reported in competitive children in comparison with healthy control groups due to significantly lower Xc/h values in absence of differences in R/h [53]. The authors suggested that it could be due to an increase in the size of the section of the limbs or to a greater ‘sufferance’ in cell membranes maybe due to bad response to the workloads (over-training). Therefore, the interpretation of Xc/h in these cases remains unresolved.

Nescolarde et al. [36] reported differences in both whole-body and localised mean Z vectors of soccer and basketball players, attributed to the different body structure between both disciplines. Soccer players presented a whole-body vector shifted to the right on the BIVA graph compared to basketball players, due to greater R/h and Xc/h. Regarding the localised vectors, soccer players showed a shift to the left of quadriceps and hamstrings vectors, due to a decrease in R/h and an increase in Xc/h. On the other hand, gastrocnemius vectors of soccer players showed a shift to the right, due to an increase in R/h and Xc/h. The muscle groups in lower-limbs were found to be symmetrical in athletes and this could be used to detect changes in hydration and/or muscular structure.

#### Short-term vector changes (<24 h after exercise)

These types of studies (Table 1) are those which currently face more difficulties, since their validity can be easily compromised, mostly because of several factors that may affect the accuracy of the measurements despite any attempts to control them. To date, two studies have investigated the vector adaptations using this type of design.

Gatterer et al. [47] analysed the short-term bioelectrical adaptations in well-trained subjects after 1 hour of self-rated intensity cycle ergometer test in the heat (environmental chamber). They reported an increase in both R/h and Xc/h after exercise, as well as significant vector migration indicating fluid loss. Besides, they pointed out a negative relationship between changes in Xc/h and in plasma osmolality (P_osm_) (r = -0.58). The authors concluded that “classic” BIVA changes mirrors water loss during exercise in the heat, and that changes in Xc/h values reflect fluid shifts between intracellular and extracellular compartments. As mentioned before, Xc is related to Cm, which is affected by the size, thickness, composition and distance between cell membranes [76]. Exercise generates processes which modify the characteristics of muscle cells (such as changes in fluid distribution). As suggested, the cell membrane becomes thinner as the cell swells and Cm increases, and the opposite happens as the cell shrinks [77], thus affecting Xc. Besides, as the cell swells, the distance to the adjacent cell membranes decreases and Cm increases (the opposite happens as the cell shrinks), also affecting Xc. Moreover, in accordance with De Lorenzo et al. [78], variations in fluid distribution would change the impedance locus and, consequently, the characteristic frequency (Fc), defined as the frequency at which Xc presents a greater value and that it is close to 50 kHz. Thus, these variations would evoke considerable changes in Xc at 50 kHz, the frequency used in BIVA [79, 80]. Nonetheless, De Lorenzo and collaborators’ hypothesis should be considered with caution because it refers to the Hanai’s model, which relays on assumptions such as spherical cell shape. Therefore, multiple factors may affect Xc values and further research should focus on this parameter in exercise.

According to Gatterer et al. [47], Carrasco-Marginet et al. [57] reported significant vector displacements along to the major axis after exercise due to significant increases in R and Xc. Furthermore, the mentioned study showed that BIVA paired graph seems to identify significant vector differences after exercise inducing mild dehydration (average loss of <1% BM) in different groups of athletes.

In opposition to both studies [47, 57], Antoni et al. [51] only found a tendency to reduction of fluids (the authors related it to an extracellular water decrease given by a significant increase in Xc) along with an increased BM in a group of men and no differences in women after approximately 10 hours of subterranean exploration (caving). Factors affecting protocols measuring pre- and post-exercise (such as dietary intake during cave activity or the skin temperature in the post measurement) could have influenced their observations. Nevertheless, despite the fact that the vector changes after fluid removal and overload (the wet–dry cycle of dialysis) as a non-physiological process is clinically well-established [69], every dehydration process induced by physical exercise is consequence of scarcely explored physiological adaptations as regard of the vector behaviour, especially at cellular level (and therefore, affecting R and Xc). In literature, Xc is an indicator of dielectric mass (membranes and tissue interfaces) in soft tissues [71]. Given the results observed in sport, it is possible that the behaviour of Xc could be due to other factors and, thus, its meaning remains to be clarified.

#### Long-term vector changes

Studies investigating long-term (≥7 days) vector adaptations (Table 2), have some protocol-specific advantages in comparison with investigations focused on acute vector changes, mainly because the quality of the bioelectrical signal can be assessed independently from the acute adaptations related to exercise.

BCM and extracellular mass (ECM) have been proposed as representatives of ICW and ECW, respectively [46]. Nevertheless, it is important to note that the estimation of fluid volumes and cell mass with BIA prediction models is inappropriate when discussing changes in vector positions after interventions or treatments. Gatterer et al. [46], in their study assessing body composition using “classic” BIVA in the 2008 European Soccer Championship, found a significant lengthening of the vector within a period between 1 and 2 weeks. They attributed it to changes in BCM and ECW in both starters and non-starters after the first match with respect to baseline values, indicating body fluid loss. After the second match, only the athletes who played more (starters) showed a significant lengthening of the vector possibly due to a decrease in ECW. Therefore, they concluded that changes in body composition were mainly due to changes in ECW. However, their results should be taken with caution, since only analysis with appropriate reference methods (e.g. isotope dilution) can support them.

Similarly to the results of Gatterer et al. [46], rapid loss of BM protocols within a few days before competition in boxers [50] was found to be achieved mainly by isotonic dehydration (they attributed it principally due to changes in ECW), as identified by the significant vector lengthening on the RXc point graph and the decreases in plasma and blood volume. Nevertheless, as mentioned before, their results should be further investigated with appropriate reference methods for the estimation of fluid volumes, since BIA prediction models are inappropriate to discuss changes in vector positions. According to the results of Reljic et al. [50], Piccoli et al. [49], also found a significant lengthening of the vector with isotonic dehydration at high altitude (5500 m). Nevertheless, although a subsequent hypertonic dehydration was identified by a decreased BM (-3.0 kg) and several hydration biochemical markers, the vector lengthening was not significant. The causes that explain why the vector remained unchanged after such a BM loss were not elucidated, and the authors recognised the difficulty of explaining the metabolic reasons that led to such BM reduction. In any case, emphasis should be placed on the importance of not considering body fluids quantitatively only (i.e., volume), but also regarding their qualitative composition, due to the biological adaptations generated by different types of exercise. For instance, after descent to sea level, the impedance vector underwent a significant shortening and returned close to baseline values. Lastly, significant relationships were found between changes in bioelectrical variables (R/h and Xc/h) and changes in the following hydration biomarkers along measurements performed at altitude and at sea level: BM, urine volume, P_osm_, serum Na^+^, K^+^, Cl^-^ and glucose, and urine osmolar excretion [49].

On the other hand, two studies [55, 56] found significant shortening of the vector along three weeks of multistage road bicycle race, indicating fluid gain during the tour and attributing these results to muscle oedema, haemodilution, released water from muscle glycogen oxidation, and excess fluid intake. Although the vector shortening was not related to power output or rating of perceived exertion [55], it was negatively associated with performance during the last stages [56], suggesting the authors that increases in plasma volume and improved thermoregulatory capacity could explain these outputs. Nevertheless, their results should be taken with caution, since measurements were performed approximately two hours after exercise and this could have altered the data.

Regarding studies analysing longer-term vector adaptations, Mascherini et al. [48] analysed a soccer team across a sport season and reported a significant shortening of the vector in the pre-season associated with an improvement in endurance performance possibly due to plasma volume expansion and enhanced glycogen storage. These results are in agreement with other studies [45, 54] which also found significant bioelectrical differences in the pre-season, hypothesising that they were due to fluid expansion. Bonuccelli et al. [45] and Macherini et al. [48] found a significant lengthening of the vector in the mid-season compared to pre-season results. This could indicate a reduced body fluid volume (i.e., decreased plasma or interstitial volume) despite an increased intracellular fluid associated with an increase in BCM, and consequently in PA [41]. However, while Mascherini et al. [48] reported a significant shortening of the vector at the end of the season compared to the mid-season, Bonuccelli et al. [45] observed a significant water content decrease. Sport calendars could have led to adopt training strategies inducing different performance status and evoked opposite vector displacements.

On the other hand, regarding the age-related decreases in Xc and PA [81], improvements have been reported after six months of resistance training in elderly women [52], suggesting increased amount and quality of soft tissues. These improvements were accompanied by increases in leg strength and thigh circumference. Along with these changes, BIVA showed a significant vector migration after the training program.

With regard to children, one study [53] evaluated the body composition in participants of swimming and gymnastics along one year. The baseline measurement (T0) was performed at a period preceding races and sporting events, just as the third measurement (T2) one year later. The second measurement (T1) was executed six months after T0 in a period characterised by a softer daily training. They found a significant increase in Xc from T0 to T1, along with increased PA and ICW (derived from ECW/TBW ratio). The authors hypothesised that this was due to an improvement in the muscular trophism with higher levels of intracellular proteins and glycogen and to a lower stress from training program. After one-year follow-up, no significant differences were found in R, Xc and PA. However, again, their hypotheses should be taken with caution, since fluid estimations were calculated from BIA prediction models. Variables as the type of sport and training strategy should be taken into account when monitoring along a season, since they might influence the bioelectrical measures. Moreover, also intra-group comparisons between seasons should be analysed with caution, since inter-seasonal bioelectrical variations could be effected by factors such as biological maturation.

#### Injury identification and follow-up

These studies [36, 37] consisted in single cross-sectional protocols aiming to identify bioelectrical patterns of change depending on the injury type and grade, and longitudinal protocols aiming at assessing bioimpedance vector sensitivity to monitor injuries and their recovery. R and Xc were found to be decreased in the injured muscles due to the oedema and to the disruption of the muscle structure, respectively. Furthermore, the more severe the injury was, the more R and Xc were decreased. On the other hand, a bioelectrical symmetry between muscular groups in lower-limbs was found. The follow-up of the injury identified bioelectrical patterns of changes similar to those in wound healing and an increase of R and Xc values were observed to values close to pre-injury.

Overall, localised bioimpedance vector analysis appears as an alternative method that could help to assess soft tissue injury and to monitor the injury recovery process [36, 37].

### Prospective research applications in sport and research agenda

BIVA in sports and exercise science is an emerging area of research with potential. The present document aims, not only to systematically overview the available scientific information, but also to outline areas of priority, future perspectives and a research agenda on this topic.

From the methodological standpoint, closely related to the quality, reliability and validity of the bioelectrical signal, some issues should be deeper investigated. For example, adequate hydration protocols are required in order to assess participants in a euhydrated state. Related to this, rigorous fluid intake control before bioelectrical measurements should be performed and reported. In studies assessing BIVA after exercise, adequate protocols of cold water application before testing with different duration and temperatures in order to reduce the sources of error in bioelectrical measurements should also be investigated, adapting the protocol to the type, intensity and duration of the exercise. Core and skin temperature should be monitored pre- and post-exercise. In sport practice, baseline values for BIVA should be established before the start of any follow-up protocol (e.g. to monitor changes along a sport competition) in the attempt to guarantee an optimal hydration status and to avoid excessive fluid loss.

Further research is also required on how much some factors affect the bioelectrical signal, especially in exercise-induced acute vector change assessment (e.g. exhaustive control of quantity and composition of fluids and food intake, and time between fluids/food intake and the bioelectrical measurements). With regard to differences in the bioelectrical signal among type of electrodes, distribution of the electrodes (e.g. whole-body standard placement or eight-polar tactile distribution), and BIA devices, further research is required. Standardisation of contact electrodes is necessary for valid BIA measurements.

As for the bioelectrical parameters, especially Xc, it will be difficult to obtain conclusions as valid and accurate as possible concerning to their patterns until the behaviour of cells in the human body is not well explained using simulated circuit models (in series, in parallel or mixed), for both homeostatic and non-homeostatic conditions. Regarding Xc changes after exercise, further research is needed in order to clarify the causes of these behaviour. As for PA, its relationship with cell functioning in sport should also be addressed.

Another critical point needing further investigation is the assessment of the validity and reliability of “classic” BIVA as a method for monitoring BCM and hydration status in sports and exercise. New specific tolerance ellipses for each sport, sex, age and race, should be generated and it should be investigated whether they can be used for the classification of an individual vector (in terms of hydration status, body composition and sport performance level) and if they represent significant hydration changes (that compromise health or performance) or target zones of impedance vectors for athletes. With regard to the hydration assessment, the analysis of the hydration status should be related to euhydration and physiological dehydration processes. In this way, as for the identification of dehydration according to the tolerance ellipses of the reference population, the limits for “normal hydration” should be reviewed. Research investigating different levels of dehydration and their relationship with the new specific tolerance ellipses is needed in order to identify the limit of “normal hydration”. Furthermore, research is needed related to the sensitivity of “classic” BIVA to each type of dehydration, as well as the behaviour of each one with regard to the tolerance ellipses. On the other hand, research investigating the relationship between the new specific tolerance ellipses and different sport performance levels is required. With regard to the body composition assessment, it should be further investigated the effect on the bioelectrical signal of the FM, fluid overload and cross-sectional areas of the body. Furthermore, future investigations should seek to clarify if BCM changes shown by “classic” BIVA mean actually BCM variations, different fluid distribution between compartments, or a combination of both. More research is needed with regard to the application of “specific” BIVA in the sports field. Comparisons of BIVA outcomes with validated body composition and fluid status assessment are to be undertaken to better define the basis for interpretation and application of this technique. These types of analyses should be undertaken in both laboratory and field conditions adjusted to the reality of sport. On the other hand, it is surprising to realise how few reliability studies in BIVA there are, this being a critical factor in establishing its practical application as a diagnostic tool.

With regard to the localised bioimpedance vector analysis, it seems necessary to standardise the distribution of the electrodes and generate muscle-specific ellipses in order to improve the reproducibility of bioelectrical measurements. This standardisation should consider the muscle length instead of the body height to normalise the bioelectrical values, since differences in the proportionality between subjects may lead to greater errors. Besides, the symmetry between limbs should be determined for each sport and discipline, particularly in relation with differences between dominant and non-dominant limbs and asymmetrical sports (e.g. jumps, throws, team sports, tennis). When speaking of localised assessment in injured muscles, further research is needed in order to establish ranges of alterations in bioelectrical vector outcomes, as well as the time course of injury recovery and return-to-play.

Regarding sports practice, PA and “classic” BIVA showed that the intense training changed functional and hydration parameters of the athletes [43]. It should be analysed if BCM and fluid content reflect the sport-specific adaptations of BM and composition. Furthermore, the utility of integrated evaluation of PA and BIVA to identify possible risks derived by different training loads in athletes should be investigated. Further research is also required to assess the relationship between BIVA and other body composition techniques.

Related tests in acute and long-term designs (e.g. muscle function, glycogen storage, haematological and biochemical markers, etc.) should be performed to correlate them with vector displacements, in order to understand better the cause of vector migration. In addition, vector changes at the medium term (< 7 days) should be investigated. Finally, it would be interesting to investigate whether the vector position is an indicator of different individual biological responses to the training load or if it is the result of optimised training activity and/or recovery strategy.

With regard to the technical requirements to perform valid measurements (see the S1 Appendix for more information), the bioimpedance assessment must be performed by using a phase-sensitive device at 50 kHz, in a room with neutral environment. The whole-body assessment has to be performed through the standard tetra-polar electrode distribution. On the other hand, the localised assessment needs a standardisation of the electrodes placement. The minimal distance between electrodes must be 5 cm and, in the case that is needed, the electrode which should be moved is the proximal one. Furthermore, before placing the electrodes, the skin must be prepared by shaving the electrode site to remove hair, rubbing with gel and cleaning with alcohol. Another important requirement is the use of appropriate contact electrodes (i.e. electrically neutral). For the assessment, the subject must be euhydrated, with no injuries or disease condition. The site of the electrodes should be changed in case that skin lesions are at the sight of the original electrodes location. The evaluation should be performed in fasting state (for at least 8 hours) and avoiding previous alcohol ingestion. Besides, the measurement should be performed once the bladder is voided and after at least 10 minutes of stabilisation. In longitudinal protocols with different measurements, the position of the electrodes has to be marked, in order to preserve the same location. Furthermore, the temperature of the skin should be controlled, in order to measure in the same conditions. The environmental characteristics should be identical between assessments. The measurement after exercise should be performed once the electrolytes of the skin have been removed with a shower and the skin temperature, cutaneous blood flow and bioelectrical parameters have stabilised to basal values. No food/drink should be consumed between measurements in the evaluation of acute variations after exercise. Nevertheless, in ecological protocols, where this condition is difficult to be followed, the quantity, moment and characteristics of the food/drink consumed should be registered. Furthermore, in ecological protocols, it should be taken into account that in the case that the measurement is performed < 1 hour after the food/drink intake, this ingestion will have a minimal effect on the impedance value. Thus, the type of exercise performed will determine the post-exercise stabilisation time and the moment at which the measurement can be made, which may be affected by the food/beverage intake during the exercise. On the other hand, with regard to the measurements in women, the menstrual cycle should be controlled and the comparison should be performed according to the cycle. Finally, the measurements should be performed at the same moment of the day, both for the comparison between subjects and for the intra-individual comparison between different assessments.

### Limitations

The main limitations derived from the literature analysis about the use of BIVA in the sport context are: 1) the difficulty of controlling multiple sources of error that may influence the bioelectrical signal; 2) the lack of tests correlating the bioelectrical signal (vector) with other variables studied in the literature; 3) the limited scientific evidence explaining the bioelectrical behaviour of human tissues induced by exercise; 4) the formulation of possible explanations for the bioelectrical behaviour of human tissues induced by exercise with inappropriate methodologies (e.g. the use of estimated fluid volumes with BIA prediction models to discuss vector variations); 5) the limited sensitivity of “classic” BIVA for the assessment of a) individual dehydration in exercise and b) two-compartment body composition; and 6) the scarcity of scientific information related to the use of BIVA in sport and exercise. Furthermore, we did not consider investigations in languages other than English, so an information bias might have existed.

## Conclusions

The main aim of this systematic review was to summarise the current knowledge on the applications of BIVA in sport and exercise. Contexts such as body composition, hydration, and other physiological and clinical conditions in physically active and trained individuals were checked.

As explored, BIVA is a relatively new technique that has a potential in sport and exercise, yet largely unexplored, especially for soft-tissue injury assessment. Regarding the assessment of hydration status through the current BIVA point graph, this is not a valid method to identify dehydration in individual athletes and a new approach is needed. On the other hand, “classic BIVA” is inconsistent in the assessment of two-compartment body composition and the vector position of athletes with regard to the reference population seems controversial in many cases. This is possibly due, between other factors, to the no consideration of the effect of cross-sectional areas. “Specific” BIVA emerges as the key to overcome this limitation.

Proper testing procedures to control factors that may affect the bioelectrical signal, as well as valid and reliable phase-sensitive measuring devices and appropriate disposables, are key to obtain more valid and precise impedance measurements. Currently, the relationship between the bioelectrical signal and physiological adaptations induced by different types of exercise remain largely unresolved, especially in how the structure and function of the cell are altered and how these affect the behaviour of R, and in particular Xc. Therefore, future research on BIVA related to sport and exercise should focus on these challenges.

## Supporting information

S1 Checklist PRISMA checklist for the current study. (DOC)

S1 Appendix BIVA methodological features. (DOCX)

## Abbreviations

BCM: Body cell mass
BIA: Bioelectrical impedance analysis
BIS: Bioelectrical impedance spectroscopy
BIVA: Bioelectrical impedance vector analysis
BM: Body mass
BMI: Body mass index
Cm: Cell membrane capacitance
DXA: Dual-energy X-ray absorptiometry
ECM: Extracellular mass
ECW: Extracellular water
ECW/TBW ratio: Extracellular / total body water ratio
FFM: Fat-free mass
FM: Fat mass
H: Body height
Hotelling’s T^2^test: Test comparing mean two group vectors
ICW: Intracellular water
Mahalanobis’ D: Multidimensional distance between a point P and the mean of a group
MF-BIA: Multi-frequency bioelectrical impedance analysis
P_osm_: Plasma osmolality
PA: Phase angle
R: Bioelectrical resistance (R/h when adjusted by height)
RXc graph: R/h vs. Xc/h probabilistic plot
SD: Standard deviation
SF-BIA: Single-frequency bioelectrical impedance analysis
TBW: Total body water
Xc: Bioelectrical reactance (Xc/h when adjusted by height)
Z: Bioelectrical impedance
Z vector: Vector yield by the RXc graph
