# Supplementary material for: Bioelectrical impedance vector analysis (BIVA) in sport and exercise: Systematic review and future perspectives
Source: PLoS One. 2018 Jun 7;13(6):e0197957. doi: 10.1371/journal.pone.0197957 (PMC5991700; doi:10.1371/journal.pone.0197957)
Supplement: S1 Appendix — (DOCX) [file pone.0197957.s002.docx]

Given the broad scope of this article, it seemed appropriate to incorporate some basic BIVA’s methodological features that enable the reader to fully understand this technology. Hence the following paragraphs shed light on data acquisition, processing and analysis, as well as graph and statistical analysis and interpretation following bioelectrical measurements.

**Bioelectrical data acquisition**

“Classic” BIVA has been performed with single-frequency, multi-frequency and BIS devices using the frequency of 50 kHz because it provides the best information at a whole-body level, as it increases the signal-to-noise ratio and decreases the frequency dependent errors and the variability of electric flow paths [1]. Furthermore, equivalence between information provided by the bioelectrical parameters at 50 kHz and that provided at other frequencies has been reported [2]. Therefore, the appropriate way to perform BIVA is using a phase-sensitive bioimpedance device (in order to measure the PA and calculate R and Xc [3, 4]) at 50 kHz. As mentioned before, the phase-sensitivity characteristic is important since non phase-sensitive instruments do not measure Xc. Therefore, studies that do not meet this requirement cannot apply BIVA. Another important requirement is the use of appropriate contact electrodes (i.e. electrically neutral) to obtain valid BIVA plots for evaluation, since vectors have been shown to be significantly affected by the type of electrode used [5]. Whole-body BIVA is performed through the standard tetra-polar electrode placement [6]. BIVA has also been used in segmental body parts, e.g. regional measurements of limbs and trunk [7], and localised muscle group measurements [8] although no standardised electrodes placement procedures exist for these techniques and there is no evidence that electrode placement different that hand-to-foot is a valid approach for application of BIVA. In the sport literature analysed, the localised approach refers to the bioelectrical analysis of body segments of the lower limb which are composed by different muscle groups. The electrodes placement described is performed putting the four electrodes in line over the muscle group that is intended to be analysed (injectors externally and sensors internally), two at the beginning and two at the end of the segment. Nevertheless, other ways to place the electrodes have been described, such as locating them at certain distance from the point of maximum pain [8, 9]. Therefore, a standardisation of the localised electrodes placement is needed.

The limitations and biases of conventional BIA are well known and have been studied across multiple literature reports [6, 10-13]. Nevertheless, multiple factors need to be taken into consideration when it comes to using BIVA in sport applications to ensure the accuracy and reliability of bioelectrical signal acquisition; particularly within protocols measuring pre- and post-exercise [14-16]. These considerations include: skin preparation [17]; hydration status [18]; consumption of food or beverage [19-22]; body position and posture during measurements [19, 23, 24]; electrode impedance [5]; position and placement modification [19, 25-27]; time of body fluid stabilisation [28]; variations in cutaneous blood flow and temperature [27, 29, 30]; skin electrolyte accumulation produced by physical exercise [29]; reproducibility of bioelectrical measurements influenced by biological intra-day [21, 22, 31] and inter-day variations [20, 31]; environmental conditions [23, 24, 30]; menstrual cycle [32-34] and injury conditions [9]. Therefore, it is crucial that bioelectrical measurements are performed following strictly the principal technical requirements. As previously mentioned, the measurements must be performed in a room with neutral environment, where no strong electrical or magnetic fields can affect the assessment. Furthermore, metallic jewellery has to be removed and the subject must avoid the contact with metal frame of bed, in order to prevent electrical interferences [11]. The minimal distance between electrodes must be 5 cm to avoid interaction between electric fields [25] and, in the case that is needed, the electrode which should be moved is the proximal one [11]. Furthermore, before placing the electrodes, the skin must be prepared by shaving the electrode site to remove hair, rubbing with gel and cleaning with alcohol in order to reduce possible interferences in the assessment [35]. For the evaluation, the subject must be euhydrated, with no injuries or disease condition which can affect the measurement. The site of the electrodes should be changed in case that skin lesions are at the sight of the original electrodes location [11]. The evaluation should be performed in fasting state (for at least 8 hours) and avoiding previous alcohol ingestion. Besides, the measurement should be performed once the bladder is voided [11] and after at least 10 minutes of stabilisation [28]. In longitudinal protocols with different measurements, the position of the electrodes has to be marked, in order to preserve the same location, due the influence of the electrode placement modification in the bioelectrical outputs [19]. Furthermore, the temperature of the skin should be controlled and the environmental characteristics should be identical between assessments. As known, the increase in the skin temperature can lead to an important decrease in R [36]. Temperature increases or decreases within the range of 1 ºC appear not to significantly affect the impedance [37] and greater differences must be avoided. Before measuring after performing exercise, a shower (as cold as tolerable) should be performed in order to reduce cutaneous blood flow and temperature and remove accumulated electrolytes, which affect the bioelectrical signal [29]. This measurement must be performed once the skin temperature, cutaneous blood flow and bioelectrical parameters have stabilised to basal values. No food/drink should be consumed between measurements in the evaluation of acute variations after exercise [11]. Nevertheless, in ecological protocols, where this condition is difficult to be followed, the quantity, moment and characteristics of the food/drink consumed should be registered. Regarding these type of protocols, it should be noted that the recent ingestion of a meal or beverage (< 1 h from the ingestion to BIA measurements) appears to be "electrically silent" and to have a minimal effect on the impedance value [38]. On the other hand, with regard to the measurements in women, the menstrual cycle should be controlled and the comparison should be performed according to the cycle, in order to minimise the effect of body fluid fluctuations caused by the female hormonal kinetics [32]. Moreover, the measurements should be performed at the same moment of the day, both for the comparison between subjects and for the intra-individual comparison between different assessments in order to minimise the effect of biological intra- and inter-day variations [20-22, 31]. More information regarding the specific recommendations for the bioimpedance analysis utilisation can be found in the ESPEN Guidelines [11].

Finally, the type of sport and/or physical exercise, time of the season, and athlete’s characteristics (age, sex, competitive level, etc.), among other factors, may dramatically determine any approach aiming to provide rigorous, valid and reliable information regarding the quality of the bioelectrical signal. In fact, although a pilot research has been published [14], we are not aware of any study in the sports field assessing the validity and reliability of “classic” BIVA as an indicator of changes in body composition and hydration status. On the other hand, two studies [39, 40] showed a lack of agreement in the assessment of two-compartment body composition between “classic” BIVA and DXA in adult and elderly. This could be mainly due to the already mentioned limitation of “classic” BIVA methodology: the no consideration of the effect of cross-sectional areas. “Specific” BIVA emerges as the key for the assessment of body composition.

**Data processing and analysis**

The fundamental advancement in recent BIA research is the use of raw impedance measurements [41]. BIA relies on the conduction of a radio-frequency electrical current through the body’s fluid (water) and electrolytes [24]. Several approaches can be used to estimate body fluid volumes using BIA. Single- and multiple-frequency impedance calculate R, Xc, or Z, and use multiple-regression equations to predict TBW or ECW and, by calculation, ICW. Bioelectrical impedance spectroscopy (BIS) couples MF-BIA with the Cole model (the mathematical model that is used most often to describe both theoretical and experimental data on skeletal muscle tissue) and mixture theory (used to model multiphase systems using the principles of continuum mechanics) to predict TBW and ECW [42]. However, SF-BIA and MF-BIA methods seem inadequate to assess hydration status because of the large variability in individual predictions of fluid volumes [43] that yield unrealistic estimates of TBW and ECW in patients with altered hydration [10]. Similarly, limitations in the application of the mixture theory in multicellular, physiological systems of the human body unfavourably limit the validity of BIS to estimate fluid volumes in adults with altered fluid status [26, 44]. At present, “classic” BIVA, phase angle and regional BIS evaluate bioimpedance data relative to statistical-based reference norms for identification of physiological perturbation and evaluation of effects of intervention. Different analytical methods have been designed to graphically display and interpret bioelectrical data in order to interpret BIVA results.

**RXc graph**

This method consists in using raw R and Xc values, standardised for body height (h), to remove the effect of conductor length, and plotting them on a probabilistic graph ⎯the so called RXc graph⎯ that yields a Z vector that has length and direction. The vector length keeps an inverse relationship with the hydration status (i.e. TBW) [45], where decreased R (shorter vector) means fluid overload and increased R (longer vector) means exsiccosis (bodily dehydration). Thus, it indicates changes in TBW and not in fluid distribution between compartments (these ones should be discussed relative to PA, which is an important advantage of BIVA). On the other hand, a migration sideways of the vector due to low or high Xc would indicate decreased or increased dielectric mass of soft tissues (membranes and tissue interfaces) [46]. The sample size and the standard deviation (SD) of R/h and Xc/h shape the size of the ellipses (i.e. the bigger the sample size, the smaller the size; and the higher the SD, the bigger the size) and the correlation between R/h and Xc/h determines the ellipsoidal form of the bivariate probability distributions: confidence intervals for average vectors and tolerance for individual vectors (i.e. the higher the correlation, the narrower the ellipse) [46].

**RXc point graph**

The individual vector or the average vector of a group could be ranked in regard to tolerance ellipses representing 50%, 75% and 95% according to the values of a given reference population [46, 47]. Besides, an individual’s bioimpedance follow-up along successive measurements can be performed with the so-called “RXc path graph” (Fig A).


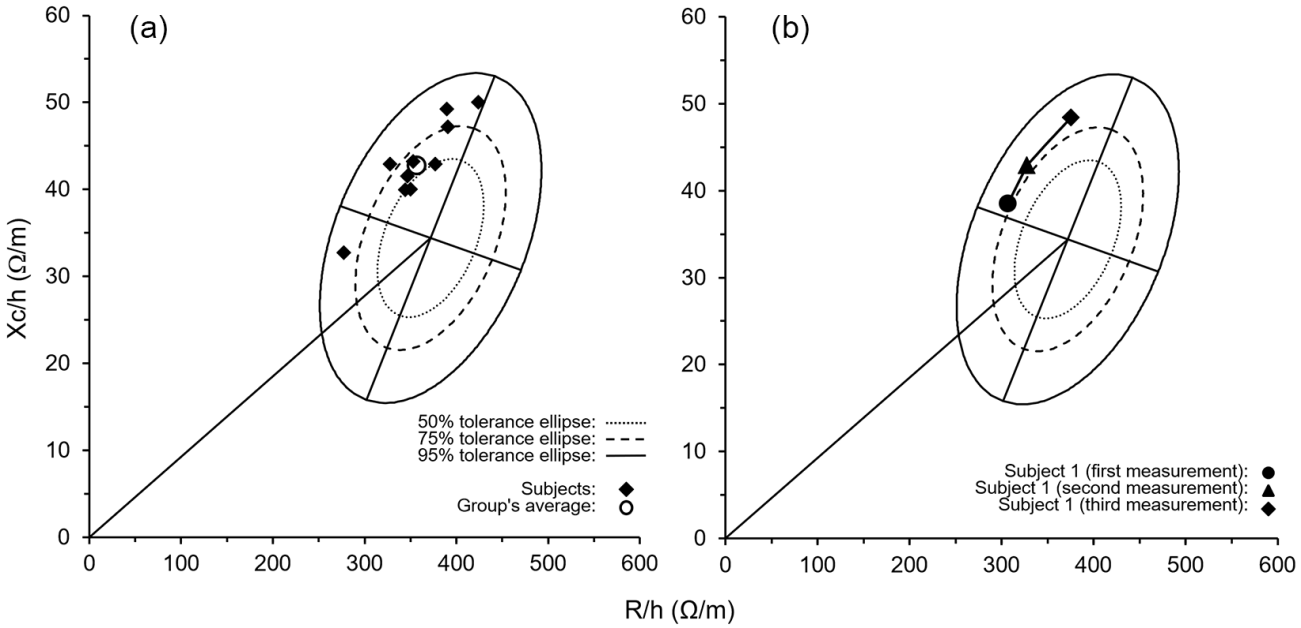


**Fig A.** **RXc point graph.** Major axis refers to tissue hydration status, and minor axis refers to soft tissue status. The vector migration grid refers to changes in both hydration and soft tissue status [46]. On the left side (a), example of standardised individual and mean impedance vectors plotted on the RXc point graph. On the right side (b), an example of an individual’s bioimpedance follow-up along successive measurements plotted on the RXc path graph. R, resistance; Xc, reactance; h, height; Ω, ohms; m, metres.

Changes in hydration status without tissue structure variations are associated with the shortening (hyperhydration) or lengthening (dehydration) of the vector in the direction of the major axis of the tolerance ellipses (normal reference for sex). Changes in mass or soft tissues structure (thin and adipose) are associated to a vector displacement in the direction of the shorter axis of the ellipses, with increased PA (obese, athletes) or a decreased PA (malnutrition/cachexia, anorexia). Combined variations of hydration and nutrition status are associated to a vector migration towards the two combined main directions [48].

**RXc score graph**

After transforming vector components into bivariate Z-scores, measurements can be compared with any populations through its standard reference intervals using the so called RXc score graph [49] (Fig B). This characteristic is especially relevant to assess how many standard deviations is an athlete or a group away from other athletes/groups. Additionally, the transformation into Z values allows the comparison of bioelectrical values measured with different devices.


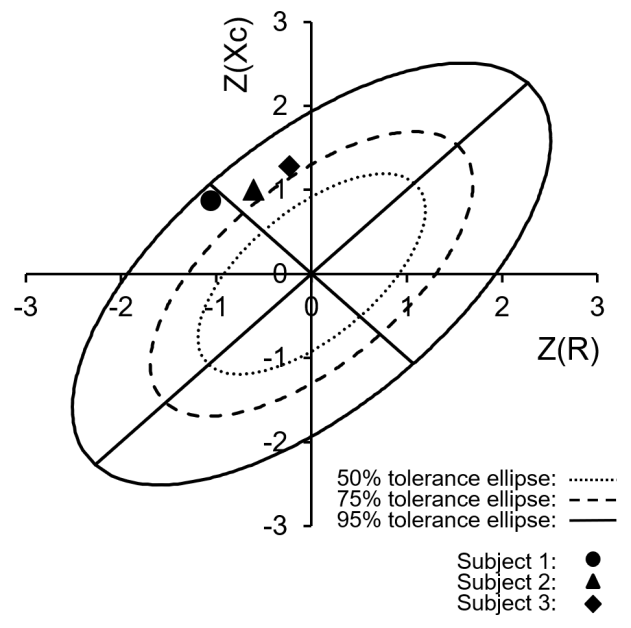


**Fig B.** **Standardised individual impedance vectors plotted on the RXc score graph.** Z(R): standard resistance score, Z(Xc): standard reactance score.

**RXc mean graph**

The mean vector of different groups of subjects can be plotted with the 95% confidence ellipse using the RXc mean graph [50] (Fig C). This graph allows to clearly visualising the PA of different groups. It also shows the differences between groups according to the shape of their 95% confidence ellipses, conditioned by their sample size, standard deviation and R/h-Xc/h correlation.


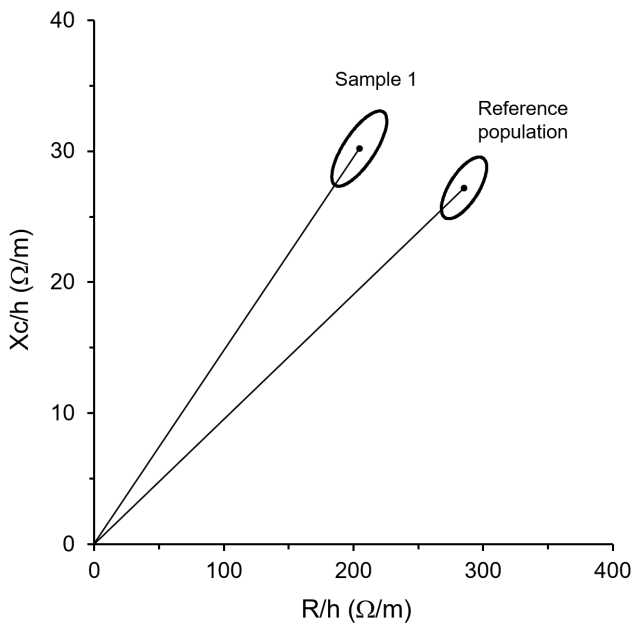


**Fig C.** **Comparative example of two mean impedance vectors plotted on the RXc mean graph: one sample (vector shifted to the left) vs. the corresponding reference population.** R: resistance, Xc: reactance, h: height, Ω: ohms, m: metres.

**RXc paired graph**

The vector displacement of a group of subjects can be plotted with the 95% confidence ellipse using the RXc paired graph [50] (Fig D). The main advantage of this graph is a clearly visualisation of the bioelectrical differences between two measurements (e.g. pre-post physical exercise protocols).


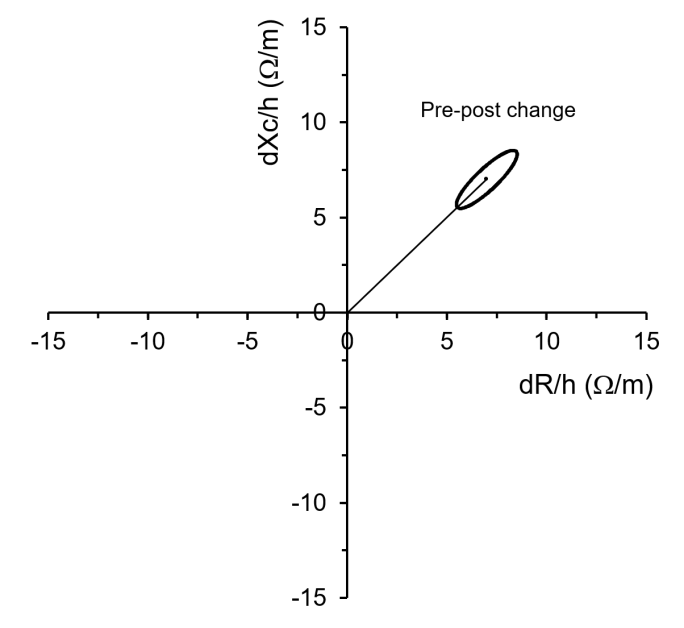


**Fig D.** **Example of pre-to-post intervention differences of a sample plotted on the RXc paired graph.** dR: resistance difference, dXc: reactance difference, h: height, Ω: ohms, m: metres.

**Phase angle (PA)**

The PA is an impedance parameter also used to overcome BIA limitations. It is measured with a phase-sensitive device and is the geometric relationship between R and Xc (expressed as the arc tangent of Xc/R) [48]. By definition, it is positively associated with Xc and negatively associated with R [51]. PA expresses the quantity and quality of soft tissue [3] and it has been suggested to be an indicator of cellular health [52, 53], where higher values reflect higher cellularity, cell membrane integrity and better cell function.

Physical activity shows a positive relationship with the PA, where subjects who perform more physical activity have higher PA, probably due to greater muscle mass [3]. This implies that higher hypertrophy levels of the skeletal muscle are related to greater PA [2] and the atrophy of the muscle mass entails lower PA [54]. Regarding the behaviour of the PA relative to dehydration, higher angles have been observed after exercise [15, 16].

Nevertheless, the use of PA alone can provide biased information. For instance, obese and athletic subjects can theoretically produce identical PA values. “Classic” BIVA allows the differentiation between these types of subjects with equal PA through the length of the vector and provides a more detailed understanding in terms of hydration status and cell mass [3].

**Overview on the statistical analysis following BIVA assessment**

**Hotelling’s T^2^ test and RXc graph**

**Unpaired data analysis**

The comparison between mean vectors from different groups of subjects is performed through the two-sample Hotelling's T^2^ test. If the 95% confidence ellipses of two mean vectors do not overlap, their position is significantly different (p <0.05). Generally, the reverse is true but not always, because there are some situations where confidence intervals overlap slightly, while Hotelling’s test still finds a significant difference at the 5% confidence level [50].

**Paired data analysis**

The analysis of the mean difference between two impedance vectors measured in two conditions in the same group of people is performed through the paired one-sample Hotelling's T^2^ test. A significant vector displacement (p <0.05) is considered if the 95% confidence ellipse of the vector does not cover the origin of the RXc paired graph. In this case, the opposite is also true, due to the use of confidence intervals of the difference in paired analysis [50].

**Mahalanobis’ generalised distance**

The Mahalanobis’ distance (D) is a scale used to distinguish among groups by means of multivariate data set analysis [55]. D is a multidimensional generalisation to measure how many standard deviations a point P is away from the mean of a given distribution. This distance is zero if P is at the mean of D, and grows as P moves away from the mean: along each principal component axis, it measures the number of standard deviations from P to the mean of the distribution, and uses within-groups variation (elliptical shape) as a yardstick for differences between means (e.g. if D = 4 between two vectors, then vectors differ by 4 within-group variation). Mahalanobis distance is unitless and scale-invariant, and takes into account the correlations of the data set.

**References**

1. Martinsen OG, Grimnes S. Bioimpedance and bioelectricity basics. London: Academic press; 2011.

2. Piccoli A, Pastori G, Codognotto M, Paoli A. Equivalence of information from single frequency v. bioimpedance spectroscopy in bodybuilders. Br J Nutr. 2007;97(1):182-92.

3. Norman K, Stobaus N, Pirlich M, Bosy-Westphal A. Bioelectrical phase angle and impedance vector analysis--clinical relevance and applicability of impedance parameters. Clin Nutr. 2012;31(6):854-61.

4. Lukaski HC, Kyle UG, Kondrup J. Assessment of adult malnutrition and prognosis with bioelectrical impedance analysis: phase angle and impedance ratio. Curr Opin Clin Nutr Metab Care. 2017;20(5):330-9.

5. Nescolarde L, Lukaski H, De Lorenzo A, de-Mateo-Silleras B, Redondo-del-Río M, Camina-Martín M. Different displacement of bioimpedance vector due to Ag/AgCl electrode effect. Eur J Clin Nutr. 2016;70(12):1401–7.

6. Yanovski SZ, Hubbard VS, Heymsfield SB, Lukaski HC. Bioelectrical impedance analysis in body composition measurement: National Institutes of Health Technology Assessment Conference Statement. Am J Clin Nutr. 1996;64(3):524S-532S.

7. Codognotto M, Piazza M, Frigatti P, Piccoli A. Influence of localized edema on whole-body and segmental bioelectrical impedance. Nutrition. 2008;24(6):569-74.

8. Nescolarde LY, J.; Medina, D.; Rodas, G.; Rosell-Ferrer, J. Assessment and follow-up of muscle injuries in athletes by bioimpedance: preliminary results. Conf Proc IEEE Eng Med Biol Soc. 2011;1137-40.

9. Nescolarde L, Yanguas J, Lukaski H, Alomar X, Rosell-Ferrer J, Rodas G. Localized bioimpedance to assess muscle injury. Physiol Meas. 2013;34(2):237-45.

10. Kyle UG, Bosaeus I, De Lorenzo AD, Deurenberg P, Elia M, Gómez JM et al. Bioelectrical impedance analysis-part I: review of principles and methods. Clin Nutr. 2004;23(5):1226-43.

11. Kyle UG, Bosaeus I, De Lorenzo AD, Deurenberg P, Elia M, Manuel Gomez J et al. Bioelectrical impedance analysis-part II: utilization in clinical practice. Clin Nutr. 2004;23(6):1430-53.

12. Dehghan M, Merchant AT. Is bioelectrical impedance accurate for use in large epidemiological studies? Nutr J. 2008;7(1):1.

13. Kushner RF, Gudivaka R, Schoeller DA. Clinical characteristics influencing bioelectrical impedance analysis measurements. Am J Clin Nutr. 1996;64(3):423S-427S.

14. Gatterer H, Schenk K, Laninschegg L, Schlemmer P, Lukaski H, Burtscher M. Bioimpedance identifies body fluid loss after exercise in the heat: a pilot study with body cooling. PloS one. 2014;9(10):e109729.

15. Antoni G, Marini E, Curreli N, Tuveri V, Comandini O, Cabras S et al. Energy expenditure in caving. PloS one. 2017;12(2):e0170853.

16. Carrasco-Marginet M, Castizo-Olier J, Rodriguez-Zamora L, Iglesias X, Rodriguez FA, Chaverri D et al. Bioelectrical impedance vector analysis (BIVA) for measuring the hydration status in young elite synchronized swimmers. PloS one. 2017;12(6):e0178819.

17. Laferriere P, Lemaire ED, Chan AD. Surface electromyographic signals using dry electrodes. IEEE Trans Instrum Meas. 2011;60(10):3259-68.

18. Berneis K, Keller U. Bioelectrical impedance analysis during acute changes of extracellular osmolality in man. Clin Nutr. 2000;19(5):361-6.

19. Gualdi-Russo E, Toselli S. Influence of various factors on the measurement of multifrequency bioimpedance. Homo. 2002;53(1):1-16.

20. Deurenberg P, Weststrate JA, Paymans I, van der Kooy K. Factors affecting bioelectrical impedance measurements in humans. Eur J Clin Nutr. 1988;42(12):1017-22.

21. Rodríguez G, Moreno LA, Sarría A, Fleta J, Bueno M. Assessment of nutritional status and body composition in children using physical anthropometry and bioelectrical impedance: influence of diurnal variations. J Pediatr Gastroenterol Nutr. 2000;30(3):305-9.

22. Slinde F, Rossander-Hulthen L. Bioelectrical impedance: effect of 3 identical meals on diurnal impedance variation and calculation of body composition. Am J Clin Nutr. 2001;74(4):474-8.

23. Rush EC, Crowley J, Freitas IF, Luke A. Validity of hand‐to‐foot measurement of bioimpedance: standing compared with lying position. Obesity. 2006;14(2):252-7.

24. Lukaski HC. Biological indexes considered in the derivation of the bioelectrical impedance analysis. Am J Clin Nutr. 1996;64(3):397S-404S.

25. Gartner A, Maire B, Delpeuch F, Sarda P, Dupuy RP, Rieu D. Importance of electrode position in bioelectrical impedance analysis. Am J Clin Nutr. 1992;56(6):1067-8.

26. Thomas B, Ward L, Cornish B. Bioimpedance spectrometry in the determination of body water compartments: accuracy and clinical significance. Appl Radiat Isot. 1998;49(5):447-55.

27. Foster KR, Lukaski HC. Whole-body impedance–what does it measure? Am J Clin Nutr. 1996;64(3):388S-396S.

28. Slinde F, Bark A, Jansson J, Rossander-Hulthén L. Bioelectrical impedance variation in healthy subjects during 12 h in the supine position. Clin Nutr. 2003;22(2):153-7.

29. O'Brien C, Young AJ, Sawka MN. Bioelectrical impedance to estimate changes in hydration status. Int J Sports Med. 2002;23(5):361-6.

30. Buono MJ, Burke S, Endemann S, Graham H, Gressard C, Griswold L et al. The effect of ambient air temperature on whole-body bioelectrical impedance. Physiol Meas. 2004;25(1):119.

31. Kushner RF, Schoeller DA. Estimation of total body water by bioelectrical impedance analysis. Am J Clin Nutr. 1986;44(3):417-24.

32. Gleichauf C, Roe D. The menstrual cycle's effect on the reliability of bioimpedance measurements for assessing body composition. Am J Clin Nutr. 1989;50(5):903-7.

33. Lusseveld E, Peters ETJ, Deurenberg P. Multifrequency bioelectrical impedance as a measure of differences in body water distribution. Ann Nutr Metab. 1993;37(1):44-51.

34. Mitchell CO, Rose J, Familoni B, Winters S, Ling F. The use of multifrequency bioelectrical impedance analysis to estimate fluid volume changes as a function of the menstrual cycle. In: Ellis K, Eastman J, editors. Human Body Composition. New York: Springer; 1993. pp. 189-91.

35. Hermens H, Freriks B, Merletti R, Stegeman D, Blok J, Rau G, et al. European recommendations for surface electromyography: Results of the SENIAM Project. Enschede: Roessingh Research and Development; 1999, pp. 1-122.

36. Caton JR, Mole PA, Adams WC, Heustis DS. Body composition analysis by bioelectrical impedance: effect of skin temperature. Med Sci Sports Exerc. 1988;20(5):489-91.

37. Liang M, Norris S. Effects of skin blood flow and temperature on bioelectric impedance after exercise. Med Sci Sports Exerc. 1993;25(11):1231-9.

38. Evans W, McClagish H, Trudgett C. Factors affecting the in vivo precision of bioelectrical impedance analysis. Appl Radiat Isot. 1998;49(5-6):485-7.

39. Buffa R, Saragat B, Cabras S, Rinaldi AC, Marini E. Accuracy of specific BIVA for the assessment of body composition in the United States population. PloS one. 2013;8(3):e58533.

40. Marini E, Sergi G, Succa V, Saragat B, Sarti S, Coin A, Manzato E, Buffa R. Efficacy of specific bioelectrical impedance vector analysis (BIVA) for assessing body composition in the elderly. J Nutr Health Aging. 2013;17(6):515-21.

41. Lukaski HC. Evolution of bioimpedance: a circuitous journey from estimation of physiological function to assessment of body composition and a return to clinical research. Eur J Clin Nutr. 2013;67(1):S2-9.

42. De Lorenzo A, Andreoli A, Matthie J, Withers P. Predicting body cell mass with bioimpedance by using theoretical methods: a technological review. J Appl Physiol. 1997;82(5):1542-58.

43. Sun SS, Chumlea WC, Heymsfield SB, Lukaski HC, Schoeller D, Friedl K et al. Development of bioelectrical impedance analysis prediction equations for body composition with the use of a multicomponent model for use in epidemiologic surveys. Am J Clin Nutr. 2003;77(2):331-40.

44. Buchholz AC, Bartok C, Schoeller DA. The validity of bioelectrical impedance models in clinical populations. Nutr Clin Pract. 2004;19(5):433-46.

45. Lukaski HC, Hall CB, Siders WA. Assessment of change in hydration in women during pregnancy and postpartum with bioelectrical impedance vectors. Nutrition. 2007;23(7-8):543-50.

46. Piccoli A, Rossi B, Pillon L, Bucciante G. A new method for monitoring body fluid variation by bioimpedance analysis: the RXc graph. Kidney Int. 1994;46(2):534-9.

47. Piccoli A. Bioelectric impedance vector distribution in peritoneal dialysis patients with different hydration status. Kidney Int. 2004;65(3):1050-63.

48. Lukaski HC, Piccoli A. Bioelectrical impedance vector analysis for assessment of hydration in physiological states and clinical conditions. In: Preedy RV, editor. Handbook of Anthropometry: Physical Measures of Human Form in Health and Disease. New York, NY: Springer New York; 2012. pp. 287-305.

49. Piccoli A, Pillon L, Dumler F. Impedance vector distribution by sex, race, body mass index, and age in the United States: standard reference intervals as bivariate Z scores. Nutrition. 2002;18(2):153-67.

50. Pillon L & Piccoli A. (2003). U.S. Patent Application No. 10/740,911.

51. Baumgartner RN, Chumlea WC, Roche AF. Bioelectric impedance phase angle and body composition. Am J Clin Nutr. 1988;48(1):16-23.

52. Mattar J. Application of total body bioimpedance to the critically ill patient. Brazilian Group for Bioimpedance Study. New Horiz. 1996;4(4):493-503.

53. Zdolsek HJ, Lindahl OA, Sjöberg F. Non-invasive assessment of fluid volume status in the interstitium after haemodialysis. Physiol Meas. 2000;21(2):211.

54. Marini E, Buffa R, Saragat B, Coin A, Toffanello ED, Berton L et al. The potential of classic and specific bioelectrical impedance vector analysis for the assessment of sarcopenia and sarcopenic obesity. Clin Interv Aging. 2012;7:585-91.

55. Mahalanobis PC. On the generalized distance in statistics. Proc Natl Inst Sci India. 1936;2:49-55.
